# Data analytics and modelling in context to determination of moisture susceptibility of reclaimed asphalt foamed bituminous mix

**DOI:** 10.1038/s41598-024-55853-5

**Published:** 2024-03-22

**Authors:** Lokesh Gupta, Rakesh Kumar, Tulika Chakrabarti, Prasun Chakrabarti, Martin Margala

**Affiliations:** 1 Department of Civil Engineering, Sir Padampat Singhania University, Udaipur, India; 2 Department of Civil Engineering, S.V. National Institute of Technology, Surat, Gujarat India; 3 Department of Chemistry, Sir Padampat Singhania University, Udaipur, India; 4 Department of Computer Science and Engineering, Sir Padampat Singhania University, Udaipur, Rajasthan 313601 India; 5 School of Computing and Lnformatics, University of Louisiana, Lafayette, USA

**Keywords:** Foamed bituminous mix, RAP, AASHTO T283, MIST, TG-2, Moisture susceptibility, Tensile strength ratio, Resilient modulus, Engineering, Mathematics and computing

## Abstract

The presence of water badly affects the moisture susceptibility of the reclaimed asphalt Foamed Bituminous Mix (FBM). The present study is mainly emphasized to assess the moisture susceptibility of reclaimed asphalt FBM, Where RAP is being incorporated as a replacement of fresh aggregates. Moisture susceptibility of the mix is evaluated in terms of tensile strength ratio (TSR) and resilient modulus ratio, subjected to different conditioning procedures namely AASHTO T283, modified IDOT, TG-2 guidelines, and MIST. Further data analytics and regression modeling are also carried out to determine the moisture susceptibility of the mix and to check the statistics among the variables. The findings show that the incorporation of RAP in the FBM improves moisture resistance. Further, FBM containing 100% RAP shows the least moisture susceptibility in terms of TSR and Mr ratio irrespective of any conditioning type. Moreover, MIST conditioning may be preferred to assess the moisture sensitivity as it simulates the field pore pressure effects. Further, mathematical analysis is carried out to predict the moisture susceptibility of mix. Adjusted R square coefficient indicates a better fit of the prediction model developed. Overall, the study may be helpful to highway professionals in analyzing the conditioning procedures and determining the moisture sensitivity of the reclaimed asphalt Foamed Bituminous Mix.

## Introduction

Moisture sensitivity is one of the most important characteristics of bituminous mix, especially when it is used as the surface course of the pavement. This is because the surface course is constantly exposed to the water. The main issue of presence of the water on the bituminous surface is that it weakens the adhesion between the aggregate particles and the binders^1,2^. This phenomenon leads to various problems such as stripping, fatigue cracking, rutting, and moisture-induced damage to the pavement^3–6^. These consequences could be even more serious in the reclaimed asphalt Foamed Bituminous Mix^7,8^. The bursting of foam bubbles is responsible for binder coating over the aggregates and therefore results in partial or improper binder coating up to a certain extent in the FBM^9^.

This challenge of proper coating over the aggregates draws attention to the moisture sensitivity level i.e. resistance against the moisture damage; as this low resistance may significantly damage the pavement layers^10–13^. In addition, TG 2 guidelines^13^ also stress and pay weightage to moisture sensitivity studies as. RAP where RAP aggregate coated with aged bitumen is referred as black rock (aggregate)^14,15^. Since this RAP is having a coating of aged binder, it could be interesting to investigate and study the moisture sensitivity analysis of RAP based foamed bituminous mix prepared using RAP as a replacement of fresh aggregates.

Moisture susceptibility studies of cold bituminous mixes like FBM containing the high RAP content are relatively newer than hot bituminous mixes and therefore less developed. Techniques determining moisture susceptibility of the cold bituminous mix are not that developed as compared to hot mix asphalt (HMA) and thus available knowledge, methods, and techniques of HMA are helping to build the research in cold mix^16,17^. Moisture damage within bituminous mix may be due to one or the combination of its characteristics and other factors such as temperature, and pore pressure in addition to traffic decrementing the binder-aggregate adhesion^18^. Some of the available methods to evaluate the moisture damage in HMA are static immersion test (AASHTO T182), boiling water test (ASTM D3625), and rotating bottle test (EN-11697-11). These methods are based on visual assessment of binder coating over the aggregates. Moreover, some other methods are based on the behavior of the mix subjected to certain moisture conditions and curing patterns such as AASHTO T283, Tunnicliff and Root conditioning, and tensile strength ratio. In addition, wheel tracking and moisture-induced sensitivity testers are also available. To the matter of selection of method choice, the one most closely associated with the field situation shall be accepted as it can simulate better results. As far as stripping is concerned, freeze–thaw cycles may be prevalent in addition to vacuumed saturation. AASHTO T 283 methods consider the effect of the freeze–thaw cycle but do not include the pore-water pressure effect produced in the fields due to traffic wheel loads whereas Moisture induced sensitivity tester (MIST) conditioning methods simulates the pore-water effects. Illinois Department of Transportation (IDOT) has altered the AASHTO T283 procedure where the consideration of freeze–thaw in the conditioning procedure is eliminated. Due to variable mechanisms, available conditioning methods differ in measuring the moisture-induced behavior of the mix. Thus, it is important to determine how precisely the moisture damage could be envisaged in simulating the field situations.

Several literatures are available to relate the moisture sensitivity studies of conventional hot bituminous mix incorporating the RAP material but exclusively the impact of RAP on the cold bituminous mix is less familiar. Nevertheless, the RAP may improve the moisture resistance than the absolute fresh aggregate since the available residual binder covers a film around the aggregates and therefore may resist the moisture damage which is even the assumed hypothesis in the study. One of the similar findings is that utilization of 50% RAP in the HMA may produce either similar or better resistance than the fresh aggregates^19^. Further Nie, 2012^20^ reported that recycled material already containing 30% RAP is considerably less resistant and therefore may be prone to pavement damage. However, it is interesting to note that Poulikakos et al.^21^ assessed both the mix type incorporating fresh aggregates and RAP on micro as well as macro-scale and found that 40% RAP-based mix is relatively more sensitive to moisture damage. In the present macro scale standard laboratory testing such moisture sensitivity is not observed. Mallick et al.^22^ studied the moisture damage of HMA using wet trafficking with a model mobile load simulator, freeze–thaw, and MIST conditioning. For the MIST conditioning, the researcher considered the temperature 60 °C, and 30 psi pressure with 5000 cycles and reported the behavior of the mix significantly influenced the consideration of accelerated loading with high temperature.

Overall, there is wide variation in the available conditioning procedure. The objective of the study is to assess and compare the moisture susceptibility determining conditioning methods namely AASHTO T283, modified IDOT, TG-2 guidelines, and MIST, with respect to Foamed Bituminous Mix prepared using RAP as replacement of fresh aggregates in terms of tensile strength ratio and resilient modulus ratio. Resilient modulus is a nondestructive test. Therefore, the ratio of resilient modulus is explored as a measure of moisture susceptibility in addition to TSR.

## Material characterization

### RAP material

**Figure 1:**
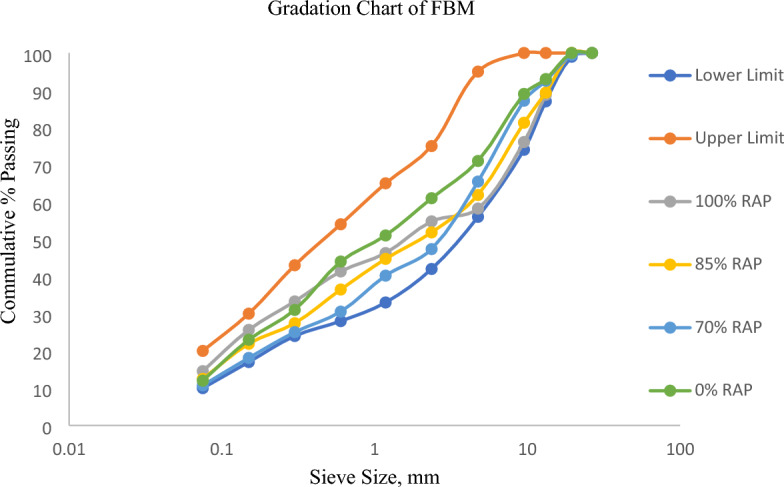
Aggregate gradation of Foamed Bituminous Mix.

The RAP material was collected in the form of large blocks. It was stockpiled for the homogeneous mix of all bags of RAP. To assess the properties of RAP, the sample was collected from about 10 places in stock as suggested in the NCHRP Report-752. West et al.^23^ and Martišius^24^ after shoveling off the top 150 mm (6 in.). The binder is extracted using a centrifugal-type bitumen extractor in accordance with ASTM D 2172/2172M-11 and obtained as 4.7% by the weight of the total mix. The physical properties of the recycled and fresh aggregates are determined in the laboratory and presented in Table 1. Gradation adopted of FBM incorporating the RAP i.e. 0% (100% fresh aggregates), 70%, 85%, and 100% with lower and upper limits (TG-2 guidelines) are presented in Fig. 1.

**Table 1:** Test results of aggregates.

SN	Particulars	Test Results		MoRTH Specifications
		Recovered Aggregate	Fresh Aggregate	
1	Aggregate impact value, (%)	25.16	23.67	Max. 30%
2	Los Angeles abrasion value, (%)	24.53	21.23	Max. 30%
3	Flakiness and elongation test, combined, %	22.47	21.42	Max. 30%
4	Water absorption, (%)	–	0.32	Max 2%
5	Specific gravity	–	2.673	–
6	Stripping, (retained coating, %)	99	96	Beyond 95%

### Homogeneity of RAP

Homogeneity of the RAP is an important factor in ensuring the quality control parameters of the cold mix incorporating the RAP. Therefore, the homogeneity of the RAP binder is assessed in terms of standard deviation and checked as per the NCHRP Report-752^23^. Table 2. Summarizes that RAP possesses acceptable homogeneity as the standard deviation of the extracted binder is within the suggested specified limits.

**Table 2:** Results of RAP binder.

Particulars	Standard deviation	Max standard deviation suggested	Reference	Test method
Binder content, %	4.7	0.5	West et al.^23^	ASTM D2172/2172M-11
Penetration, 0.1 mm @ 25 °C	2.84	4	Martišius^24^	ASTM D5-05A
Softening point, °C	1.51	2		ASTM D36-95(2000)

### Foamed bitumen

**Figure 2:**
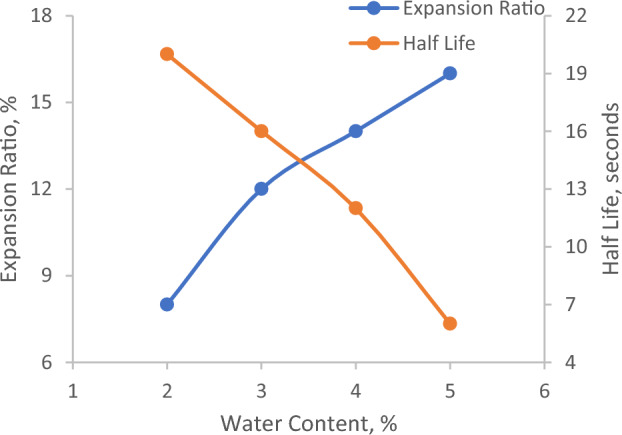
Variation in expansion ratio and half-life.

The foaming of the bitumen is achieved by injecting the water at a rate of 2% (by the weight of the binder) at 160 °C. The optimum content of water required to form the acceptable foaming characteristics is determined based on the expansion ratio and half-life. Half-life and expansion ratio are inversely proportional. With an increase in water content, the half-life decreases whereas the expansion ratio increases^13,25^. Past studies and guidelines^13,25–27^ have recommended a minimum half-life and expansion ratio of 6 s and 8% respectively. The optimum water content is evaluated as 3.5% by taking the average water content satisfying the minimum half-life and expansion ratio criteria. The variation in half-life and expansion ratio with respect to water content is plotted in Fig. 2.

## Experimental investigation

### Moisture content

**Figure 3:**
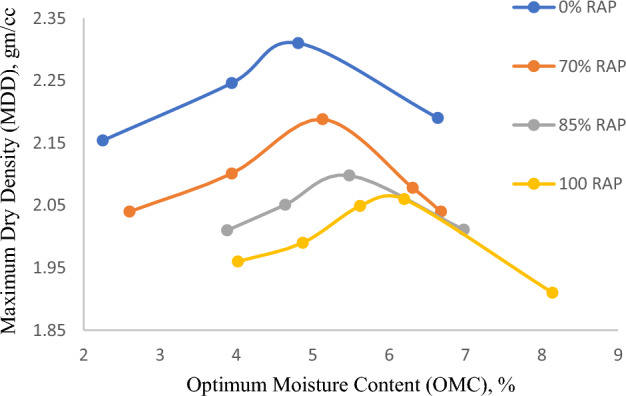
Moisture density relation curve.

The optimum moisture content (OMC) of the blended RAP material is determined as per the AASHTO T180 using the density-moisture content relationship. Loose material is mixed well by adding the water content in each trial. The density achieved corresponding to each moisture content is determined and plotted in Fig. 3. Optimum moisture content (OMC) is evaluated corresponding to maximum dry density (MDD). 65% of OMC is adopted as mixing moisture content (MMC) as per the TG-2 guidelines. This MMC is required to produce a dispersion of foamed binder within the mix.

It can be noticed from Fig. 3 that with the increase in moisture content, dry density increases up to a certain extent initially and then decreases. This trend is even true for all the combinations of RAP content. However, the rise in RAP material (0% to 100%) results in the reduction of the maximum dry density (MDD) and increase in the corresponding optimum moisture content. This increase in OMC and reduction in MDD (with the increase in the RAP content) could be due to a combination of one or more reasons such as weak bonding between the fresh aggregate and RAP, less fine material that fills the voids during compaction, or the higher flakiness and elongation combined index. Overall results in poor interlocking and thus makes the material less dense. Further increment of RAP content is accountable for the increase in the particle surface area, causing the rise in OMC.

### Preparation of foamed bituminous mix

One of the objectives of mix design is to develop the job mix formula that includes the information such as optimum binder content that is required to prepare the mix specimen. In this regard, some guidelines and literature i.e., Wirtgen^25^; Technical Guidelines^13^; Austroroads^28^ are available. Most of this literature specifies the determination of optimum foamed bitumen content (OFBC) but varies in terms of other parameters such as compaction and curing descriptions. Modarres and Ayar^29^; Kim et al.^30^ have suggested the Marshall method of compaction in association with maximum dry density to determine OFBC but literature such as Kar et al.^11^; Loizos and Papavasiliou^31^ TG-2 guidelines^13^, stress to find OFBC based on performance test like indirect tensile strength test, resilient modulus or moisture susceptibility. In the present study, OFBC is determined considering the indirect tensile strength (ITS) satisfying the criteria of minimum 100 kPa and 225 kPa tensile strength under dry and wet conditions respectively^11,13^.

Therefore, the 100 mm diameter Marshal mould is prepared by applying 75 blows on either side. The compacted mix specimen is cured at 40 °C for the duration of 72 h. Now, ITS dry is determined at 25 °C while ITS wet is evaluated after the additional conditioning of the specimen at 25 °C for 24 h post-dry curing. 3% is the minimum foamed bitumen content meeting the defined strength criteria of 100 kPa and 225 kPa in dry and wet conditions respectively.

## Methodology

Moisture sensitivity of the FBM containing the RAP subjected to different conditioning (explained in this section) is assessed in terms of tensile strength ratio (TSR) and resilient modulus (Mr) ratio. TSR and Mr ratio is determined in percentage by taking the ratio of respective ITS and Mr values of the specimen tested under wet conditioning to dry conditioning i.e. ITS wet to ITS dry and Mr wet to Mr dry respectively.

### Moisture conditioning

AASHTO T 283 and IDOT modified AASHTO T-283 (2011), MIST, and TG-2 guidelines are adopted in the present study to assess the moisture resistance potential of FBM incorporating RAP material. As far as AASHTO conditioning procedures are concerned, AASHTO T283 includes the freezing and thawing effect whereas the IDOT method does not. The conditioning procedure of both methods is discussed in Table 3.

**Table 3:** AASHTO T 283 and IDOT modified AASHTO T 283 conditioning procedure.

Conditioning	T 283	IDOT
Dry	Cover the sample in saran paper Keep the sample in the water bath at 25 °C for a duration of 02 h	Keep the unwrapped sample in the water bath at 25 °C for a duration of 02 h
Wet	Sample saturation—70% to 80% Cover the sample in saran paper and seal it in a plastic bag with 10 ml of water Freeze the sample for 16–24 h at − 18 °C Keep the sample after removing all the cover and paper in the water bath at 60 °C for a duration of 24 h Followed by 25 °C conditioning for the next two hours	Sample saturation—70% to 80% Keep the sample in the water bath at 60 °C for a duration of 24 h Followed by 25 °C conditioning for the next two hours

For moisture-induced sensitivity tester (MIST) conditioning the FBM specimen was prepared at optimum binder content and subjected to pore pressure in the chamber. MIST works on the principle of cyclic pore in combination with high water temperature to simulate the field moisture damage due to traffic and moisture simultaneously. FBM specimens prepared using RAP were subjected to 3500 cycles at a hydrostatic pressure of 40 psi (276 kPa) and 50 °C temperature. As far as TG-2 conditioning is concerned, the specimen was cured at 40 °C for 3 days and allowed to cool before the samples were then kept in the water bath at 25 ± 1 °C for 24 h. Samples are now removed from the water bath and allowed to stand on a draining board at 25 °C ± 1 °C for 30 min to determine the ITS soaked i.e. ITS wet.

For each conditioning type i.e., AASHTO T 283 (T 238) and IDOT modified AASHTO T-283 (IDOT), TG-2, and MIST, a set of three samples is tested under wet and dry conditions to determine the indirect tensile strength, resilient modulus. Subsequently, the TSR and Mr ratio are evaluated.

### Test method

Moisture sensitivity of the FBM containing the RAP subjected to different conditioning types is assessed by conducting the resilient modulus test and indirect tensile strength test in the laboratory. Resilient modulus is a nondestructive method that demonstrates the important parameter to analyze the pavement material, pavement analysis, and design. The resilient modulus test is conducted as per AASHTO D 4123-82(2009) at 25 °C after conditioning the samples to analyze the moisture sensitivity of the mix in terms of the Mr ratio. Resilient modulus is measured by applying repetitive compressive load haversine waveform with a pulse width of 0.01 s, rest period of 01 s, and poisons ratio of 0.35.

An indirect tensile strength test is performed on compacted cylindrical specimens in accordance with ASTM D6931 (2012) at 25 °C test temperature. The ultimate load sustained by the specimen before failure is measured by applying the compressive load at a constant deformation rate of 50.8 mm per minute on the diametrical axis of the specimen and indirect tensile strength (ITS) of the mix under two moisture conditions i.e., dry and soaked is evaluated using the following Eq. (1).

1 $$ {\text{ITS}} = {\text{ 2P/}}\uppi {\text{dt}} $$

where, P = Ultimate load, N; d = diameter of the specimen, mm; t = thickness of the specimen, mm.

Moisture sensitivity of the FBM containing the RAP subjected to different conditioning types i.e. ASHTO T 283 (T 238) and IDOT modified AASHTO T-283 (IDOT), TG-2, and MIST; is assessed in terms of tensile strength ratio (TSR) and resilient modulus ratio by taking the ration of respective value under wet soaked to dry conditioned specimen i.e. ITS wet to ITS dry and Mr wet to Mr dry.

## Performance evaluation

**Figure 4:**
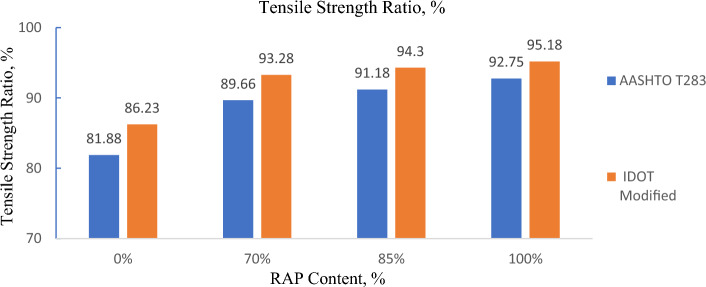
TSR results of FBM specimen subjected to AASHTO T 283 and IDOT modified.

**Figure 5:**
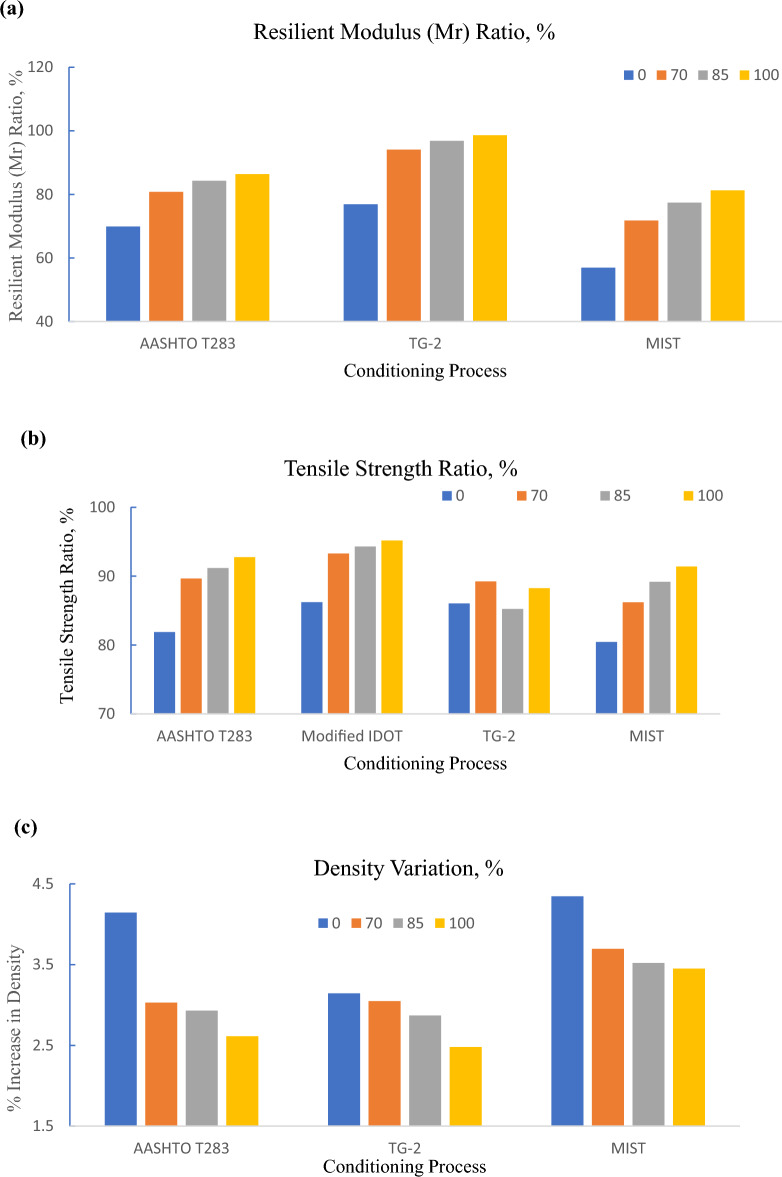
Moisture sensitivity results of FBM incorporating RAP subjected to different conditioning processes: (**a**) tensile strength ratio, %; (**b**) resilient modulus ratio, %; (**c**) density variation, %.

The section mainly emphasized studying the moisture sensitivity of Foamed Bituminous Mix prepared using reclaimed asphalt pavement material (RAP) and virgin aggregates using different conditioning types i.e. AASHTO T283, modified IDOT AASHTO T283, TG 2 guidelines, and moisture induces sensitivity tester (MIST). Further, the moisture sensitivity which is a measure of resistance against moisture damage is assessed in terms of resilient modulus (Mr) ratio and tensile strength ratio (TSR). Where Mr ratio and TSR is the percentage ratio of respective resilient modulus value and indirect tensile strength of the samples tested under wet conditioning to dry conditioning. To check the variation in the density due to changes in conditioning type, the weight of the specimen is observed before and after each and every conditioning type. Resilient modulus, density and indirect tensile strength of the specimen subjected to dry and wet conditioning are summarized in Tables 4 and 5. Furthermore, the results of Mr ratio, TSR and percentage increase in density are presented in Figs. 4 to 5 respectively.

**Table 4:** Resilient modulus ratio and % increase in density of FBM subjected to conditioning type.

RAP Content, %	Resilient modulus (Mr), MPa						Density, gm/cc					
	AASHTO T 283		TG-2		MIST		AASHTO T 283		TG-2		MIST	
	Dry	Wet	Dry	Wet	Dry	Wet	Dry	Wet	Dry	Wet	Dry	Wet
0	5368	3751	5217	4011	5112	2911	2.34	2.437	2.29	2.362	2.277	2.376
70	4217	3407	4214	3964	4652	3339	2.013	2.074	2.132	2.197	2.11	2.188
85	2992	2523	3216	3114	3834	2969	1.945	2.002	2.014	2.072	2.017	2.088
100	2568	2219	2714	2676	3466	2816	1.913	1.963	1.972	2.021	1.97	2.038

**Table 5:** Indirect tensile strength of FBM subjected to conditioning type.

RAP content, %	Indirect tensile strength, kPa							
	AASHTO T 283		IDOT modified		TG-2		MIST	
	Dry	Wet	Dry	Wet	Dry	Wet	Dry	Wet
0	414	339	436	376	423	364	404	325
70	387	347	402	375	391	349	370	319
85	363	331	386	364	373	318	351	313
100	345	320	353	336	341	301	337	308

### AASHTO T283 and IDOT

It can be noticed from Fig. 4 that IDOT modified method displays a better tensile strength ratio than the AASHTO T 283 method irrespective of any combination of FBM incorporating RAP i.e., 100%, 85%, 70%, and 0% (100% virgin aggregates). The high TSR ratio of this mix subjected to IDOT conditioning type indicates a better resistance against moisture damage. This may be due to the modified IDOT method doesn’t include the freeze–thaw cycling effect as compared to the T283 method in the conditioning procedure. Thus, might result in a higher TSR value. Knowing the fact that some regions experience prevailing winter conditions, modified IDOT results may therefore mislead or misjudge the true scenario. Hence modified IDOT method is dropped for any further conditioning type and only AASHTO T283 is preferred over modified IDOT method.

### AASHTO T283 conditioning procedure

It can be noticed from Fig. 5a and b that at 0%, 70%, 85%, and 100% RAP content, the Mr ratio of the FBM prepared using 0%, 70%, 85% and 100% RAP is 69.87%, 80.79%, 84.32% and 86.4% respectively, whereas tensile strength ratio (TSR) is 81.88%, 89.66%, 91.18% and 92.75% the respectively. FBM prepared using 100% RAP exhibits maximum resistance against moisture damage whereas, a mix containing 0% RAP (100% fresh aggregates) shows showing least resistance in terms of both Mr ratio and TSR among all other combinations of RAP. However, Mr and the TSR value is beyond 80% in all cases.

This may be attributed to any RAP binder coating left over the aggregates may be responsible for enhancing the moisture resistivity of the mix. Further, the resistance against the moisture damage of the FBM is increasing with the increase in RAP content. There is marginal variation within the TSR value and Mr ratio of the FBM mix prepared using 70%, 85%, and 100% RAP material. Overall FBM containing RAP material is less susceptible to moisture damage and subsequently possesses higher TSR value and Mr ratio than the mix prepared using fresh aggregates.

Further, Fig. 5c shows the variation in the density value of the FBM containing RAP. Water absorption may be responsible for this change in the density value. The change in density value is maximum in the case of 0% RAP of about 4.145% compared to FBM containing 70%, 85%, and 100% RAP i.e., 3.03%, 2.93%, and 2.61% respectively. This density variation is least in the case of FBM containing 100% RAP mix exhibits less water absorption characteristic of the mix that further may be attributed to higher resistance against moisture damage.

### TG 2 conditioning procedure

It can be attested from the results of the resilient modulus ratio presented in Fig. 5b that the FBM mix prepared using 100% RAP does not exhibit any moisture effect as the Mr ratio is nearly 100% i.e., 98.60%. Since Mr ratio of 70% and 85% RAP based, the mix is 94.06% and 96.82% respectively, it can be drawn that Not only 100% RAP, but FBM prepared using 70% RAP and 85% RAP also does not seem like affected by the moisture conditioning. However, results do not follow this trend in the case of MIST and or AASHTO T283 conditioning process. Mr ratio for any respective combination of RAP is relatively high in the case of TG-2 as compared to the other two types of conditioning (MIST and AASHTO T283) indicating lesser moisture damage.

It can be observed from Fig. 5c that the percentage increase in density of the specimen subjected to TG 2 conditioning is relatively lesser than AASHTO T283 and MIST conditioning indicating lesser water absorption. This trend of density variation might result in lesser moisture damage in the case of the TG 2 conditioning process. Further, as far as moisture sensitivity is concerned, resistance against moisture damage (TSR and Mr ratio both) is increasing with the increase in RAP content except for the TSR results subjected to TG 2 conditioning procedure (TSR variation is like zigzag pattern; Fig. 5a. It may be due to variability in the foam bulk mix. Further, the indirect tensile strength test is also destructive in nature where the same sample cannot be reused for testing before and after the conditioning. Considering the observations, it is quite tough to identify and confirm any significant moisture damage in the case of FBM mix containing RAP subjected to TG-2 conditioning except 0% RAP that doesn’t possess RAP binder. Therefore, in such cases, the Mr ratio may also be considered over the TSR to assess the moisture susceptibility of the mix.

### MIST conditioning procedure

Table 5 displays that, the density of the mix specimen subjected to the MIST conditioning procedure is increasing after wet conditioning. This increase in the density may be due to the absorption of water in the air voids during the conditioning process. The percentage increase in the density of the specimen subjected to the MIST conditioning procedure is maximum as compared to other conditioning procedures i.e., AASHTO T283 and TG 2 (Fig. 5c).

It can be noticed from Fig. 5 that, for every conditioning procedure (i.e. AASHTO T283, TG 2, MIST) the FBM prepared using 0% RAP is having maximum percentage increase in the density and therefore subsequently results in the least resistance against the moisture damage (Least TSR and Mr ratio). With the increase in the RAP content, the Percentage increase in the density is decreasing and hence resistance against moisture damage is increasing in terms of both TSR and MR ratio values. However, the specimen subjected to MIST conditioning procedure showed the least TSR and Mr value i.e. resistance against moisture damage as compared to other conditioning procedures (AASHTO T283 and TG 2).

This may be due to the MIST conditioning procedure simulating the field pore pressure effects that develop in the pavement under repetitive traffic. It develops as the wheels pass pushing the moisture or water to enter the voids and then releasing to come at the surface when the wheels move away. Therefore, the MIST conditioning procedure seems more reliable as it includes field conditions as compared to other conditioning types.

## Moisture susceptibility regression model

A multiple regression analysis using SPSS software is carried out to evaluate and predict the moisture susceptibility of the reclaimed asphalt Foamed Bituminous Mix.

Moisture susceptibility regression prediction model is developed for the FBM subjected to MIST conditioning type, since it is found in the discussion that MIST conditioning is relatively reliable as compared to other conditioning procedures due to its simulating effect of field pore pressure. Indirect tensile strength (Dry ITS), resilient modulus (Dry Mr), dry density, wet density and RAP content are the predictors to predict the moisture susceptibility of the FBM in terms of Mr ratio. Mr ration is a measure of moisture susceptibility representing the better resistance against the moisture damage with the higher value of Mr ratio.

### Data normality and correlation

In the analysis, data is assumed to be normally distributed and checked considering the values of skewness and kurtosis distribution. The results of the normality checked are presented in Table 6. It can be noticed from Table 6 that the skewness and kurtosis are in the range of -2 to 2 and therefore represent the normal distribution.

**Table 6:** Statistics of normal distribution check.

Parameters	Mean	Std. deviation	Skewness	Kurtosis
Rap_Content	63.75	39.299	− 0.971	− 0.745
Dry_ITS	368.35	26.448	0.408	− 1.048
Dry_Mr	4307.70	673.013	0.013	− 1.755
Dry_Density	2.0950	0.12107	0.652	− 1.106
Wet_Density	2.1750	0.13348	0.682	− 1.092
Mr_Ratio	72.2725	9.69764	− 0.792	− 0.945

The coefficient of correlation among the variables is presented in Table 7. The results of correlation coefficient shows that predictors have the direct effect on the moisture susceptibility. The dependent variable Mr ration (Measure of moisture susceptibility) is positively corelated with the RAP content i.e. increase in the RAP content enhance the resistance against the moisture damage of reclaimed asphalt FBM, whereas the other predictors i.e. Dry ITS, Dry Mr, Dry density, Wet density are negatively correlated with the moisture susceptibility.

**Table 7:** Coefficients correlation matrix.

Parameters	Correlation coefficient with Mr
Mr_Ratio	1
Rap_Content	0.993
Dry_ITS	− 0.96
Dry_Mr	− 0.92
Dry_Density	− 0.996
Wet_Density	− 0.996

### Prediction model

A multiple regression equation to predict the moisture susceptibility in terms of Mr ratio is prepared in the form of-

2 $$ {\text{M}} = {\text{B}}_{0} + {\text{B}}_{{1}} {\text{R}}_{{\text{p}}} + {\text{B}}_{{2}} {\text{T }} + {\text{ B}}_{{3}} {\text{R }} + {\text{ B}}_{{4}} {\text{D}}_{{\text{d}}} + {\text{ B}}_{{5}} {\text{W}}_{{\text{d}}} $$

where: M = moisture susceptibility in terms of Mr Ratio, %, R_p_ = RAP Content, %, T = indirect tensile strength, MPa, R = resilient modulus, MPa, D_d_ = dry density, gm/cc, W_d_ = wet density, gm/cc.

The summary of the mathematical model prepared using SPSS to predict the moisture susceptibility of the FBM is presented in Table 8.

**Table 8:** Summary of moisture susceptibility regression model.

Model	R	R^2^	Adjusted R^2^	Std. error of the estimate	Change statistics				
					R square change	F change	df 1	df 2	Sig. F change (p)
Multiple regression model	0.991	0.983	0.966	0.0061	0.983	1617.99	5	14	0
Durbin-Watson: 1.981									
a. Predictors: (constant), temperature									
b. Dependent variable: ITS									
M = 254.481 + 0.011 R_p_ + 0.005 T + 0.003 R − 111.059 D_d_ + 15.977 W_d_									

The coefficient of correlation and adjected coefficient of determination (Adjusted R^2^) are 0.991 and 0.966 respectively. Adjected R^2^ explains an extent of 96.6% variation can be explained by the predictors in determining the moisture susceptibility of the mix. Further analysis also indicates that predictors are statistically significantly associated with the dependent variable moisture susceptibility as p-value (p = 0.0000) is less than the predetermined significance level i.e. 0.05 (5%).

The Durbin Watson (DW) statistic is a test for autocorrelation in the residuals from a statistical model or regression analysis. The Durbin-Watson statistic has a value ranging between 0 and 4. Table 8 represents the value of Durbin Watson coefficient as 1.981 very close to the value of 2.0 indicating there is no autocorrelation detected in the residuals.

**Figure 6:**
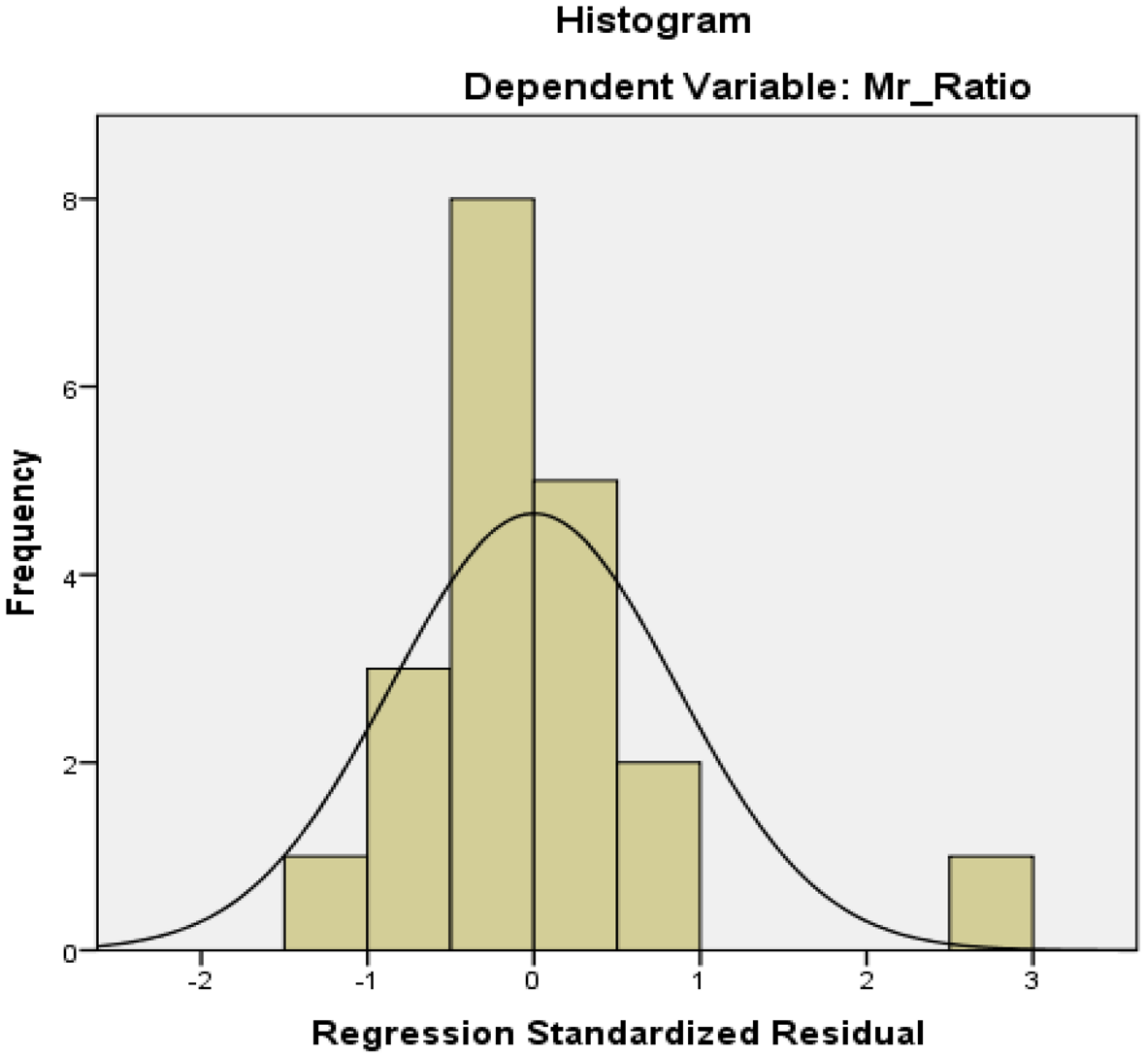
Histogram showing the normal distribution of residuals.

Figure 6 represents a histogram a plot of residuals for the dependent variable Mr ratio. In the histogram, a bell-shaped symmetrical curve is observed having a maximum score in the middle and less at the edges confirming the normal distribution of the residuals. This is one of the assumptions for the multiple linear regression analysis of the data especially when the data is less.

## Conclusions

The present study is mainly emphasized to measure the moisture susceptibility of reclaimed asphalt Foamed Bituminous Mix subjected to different conditioning procedures namely AASHTO T283, modified IDOT, TG-2 guidelines, and MIST. The outcomes of the study are summarized as follows.

- AASHTO T283 includes a freeze–thaw cycle whereas the modified IDOT method eliminates this freeze–thaw cycling effect in its conditioning procedure and therefore mislead or misjudge the true scenario in the prevailing winter conditions.
- To assess the resistance against moisture damage, MIST conditioning may be preferred as it simulates the field pore pressure effects. However, in the region that experiences winter conditions, either AASHTO T283 or MIST conditioning may be preferred considering the prevailing conditions.
- Since the TSR is a destructive test in nature, it may influence the results whereas resilient modulus is nondestructive and hence displays the better scenario of moisture damage. Further there is also possibilities that there may be the disparity in the TSR results due to variability in the foam bulk mix. Therefore, the resilient modulus (Mr) ratio may be preferred over the TSR to evaluate the moisture susceptibility of the FBM.
- Data analysis shows that predictors adopted in the study are statistically significantly associated with the dependent variable moisture susceptibility as p-value is less than the predetermined significance level. Adjested R square coefficient indicates a better fit of prediction model developed. Further, the Durbin Watson (DW) statistic also represents that there is no autocorrelation detected in the residuals.

Overall, FBM containing 0% RAP i.e., mix prepared using 100% fresh aggregates shows least resistance and maximum moisture damage in terms of TSR and Mr ratio irrespective of any conditioning procedure. Further, the incorporation of RAP material in the Foamed Bituminous Mix improves moisture resistance than the absolute fresh aggregate.

## Data Availability

All data, models, and code generated or used during the study appear in the submitted article.

## Abbreviations

FBM: Foam bituminous mix
HMA: Hot mix asphalt
ITS: Indirect tensile strength
MDD: Maximum dry density
MMC: Maximum moisture content
MORTH: Ministry of road transport and highways
Mr: Resilient modulus
OMC: Optimum moisture content
OWC: Optimum water content
RAP: Reclaimed asphalt pavement
TSR: Tensile strength ratio
